# Endothelial progenitor cell number is not decreased in 34 children with Juvenile Dermatomyositis: a pilot study

**DOI:** 10.1186/s12969-017-0171-3

**Published:** 2017-05-17

**Authors:** Dong Xu, Akadia Kacha-Ochana, Gabrielle A. Morgan, Chiang-Ching Huang, Lauren M. Pachman

**Affiliations:** 1 Cure JM Program of Excellence in Juvenile Myositis Research at Stanley Manne Children’s Research Institute of Ann and Robert H., Lurie Children’s Hospital of Chicago, Chicago, IL USA; 2 Department of Pediatrics, Division of Rheumatology, Ann and Robert H. Lurie Children’s Hospital of Chicago, Northwestern University Feinberg School of Medicine, Chicago, IL USA; 3 Zilber School of Public Health, University of Wisconsin at Milwaukee, Milwaukee, WI USA

**Keywords:** Juvenile Dermatomyositis (JDM), Endothelial Progenitor Cell (EPC), Fluorescence Activated Cell Sorting (FACS)

## Abstract

**Objective:**

A pilot study to determine endothelial progenitor cells (EPC) number in children with Juvenile Dermatomyositis (JDM).

**Methods:**

After obtaining informed consent, the EPC number from 34 fasting children with definite/probable JDM at various stages of therapy—initially untreated, active disease on medication and clinically inactive, off medication—was compared with 13 healthy fasting pediatric controls. The EPC number was determined by fluorescence activated cell sorting (FACS), CD34^+^/VEGFR2^+^/CD45dim^−^, and assessed in conjunction with clinical variables: disease activity scores (DAS), duration of untreated disease (DUD), TNF-α allelic polymorphism (A/G) at the promoter region of −308, number of nailfold capillary end row loop (ERL) and von Willebrand factor antigen (vWF:Ag). Correlations of the EPC numbers with the clinical and demographic variables, including DAS Skin (DAS SK), DAS Weakness (DAS WK), DAS Total Score, DUD, Cholesterol, triglycerides, High-Density Lipoprotein (HDL) and Low-Density Lipoprotein (LDL), and ERL were calculated using the Pearson correlation coefficient. Tests of associations of EPC with gender (boy vs girl), TNF-α-308A allele (GA/AA vs GG), vWF:Ag (categorized by specific ABO type) as normal/abnormal were performed, using two-sample T- tests.

**Results:**

The EPC number for JDM was not significantly different from the healthy controls and was not associated with any of the clinical or cardiovascular risk factors tested.

**Conclusion:**

The EPC for JDM were in the normal range, similar to adults with DM. These data support the concept that the normal EPC numbers in DM/JDM, irrespective of age, differs from adult PM, where they are decreased, perhaps reflecting a different pathophysiology.

## Introduction

Children with Juvenile Dermatomyositis (JDM) have systemic vasculopathy [1] and manifest the characteristic skin involvement, with or without symmetrical proximal muscle weakness [2]. Disease activity can be reliably evaluated by disease activity scores (DAS) [3], and is accompanied by destruction of nailfold capillary end row loops (ERL) [4]. Autoimmune vascular damage in the inflammatory myopathies is attributed, in part, to endothelial cell damage implemented by proinflammatory cytokines and chemokines, such as Type 1 interferons –IFN-β > IFN-α [5]. However, the elements of the vasculopathy in JDM remains poorly understood and the role of endothelial progenitor cells (EPCs) has not been defined in these children. EPCs were first isolated two decades ago from human peripheral blood [6]. These cells can differentiate into mature endothelial cells, which may be incorporated into sites of active angiogenesis and may participate in repair of damaged vascular endothelial cells [6]. The majority of EPCs are derived from bone marrow and may be mobilized to enter the blood stream by chemokines or angiogenic growth factors [7]. There are two major methods to identify EPC by fluorescence activated cell sorting (FACS). The first uses both CD34^+^ and VEGFR2^+^ biomarkers because: a) VEGFR2 is only present on endothelial linage cells such as endothelial progenitor cells and mature endothelial cells; b) CD34 is a marker of stem cells, and c) these double positive cells have the capacity for tube formation [6]. The second method uses CD34^+^/CD133^+^ biomarkers that are less specific for EPCs; CD133 is a stem cell marker that is not only present on immature endothelial progenitor cells, but is also found on other cell types such as epithelial cells and cancer stem cells. Of note, EPCs identified by CD34^+^ and CD133^+^ double positive have not been proven to undergo tube formation, either in vitro or in vivo [8]. With respect to other related rheumatic diseases, EPC number and functionality is damaged in adults with Polymyositis (PM), but not in adults with Dermatomyositis (DM) [9]; EPCs are impaired in adults with Systemic Lupus Erythematosus (SLE) [10] as well as adult Rheumatoid Arthritis (RA) [11]. It is not known if the EPC number and functionality is impaired in JDM. One factor that potentially might contribute to a decreased EPC number in JDM is that IFN-α, which is increased in JDM sera, has been shown to be biologically active [12]. The availability of an adequate number of the EPCs may be of importance; it is speculated that EPCs might participate in repairing the characteristic microvasculopathy of JDM [1, 4]. The purpose of this cross-sectional pilot study was to evaluate EPCs number in fasting children with definite/probable JDM at various stages of disease activity.

## Materials and methods

### Patient population

After obtaining age-appropriate informed consent, a convenience sampling of blood was obtained from 34 fasting children with definite/probable JDM [2], (IRB# 2008–13,457) as well as from 13 fasting age, race and gender matched healthy controls (IRB# 2001–11,715). Peripheral blood was drawn in trisodium citrate anticoagulant and used for mononuclear cell (PBMC) isolation. Table 1 displays demographic data for controls and JDM. Methods for the clinical variables – DAS, duration of untreated disease (DUD), determination of TNF-α alleles at the promoter region of −308; measurement of ERL number and von Willebrand factor antigen (vWF:Ag) – have been reported previously [13]. In addition, the association of EPC number with lipids — high-density lipoprotein (HDL), low-density lipoprotein (LDL), cholesterol, triglycerides, age, gender and blood type — were tested.

**Table 1:** Demographic data of children with JDM

	Boy	Girl	Mean age	White	Hispanic	Asian	Other
Control	2	11	11.5+/−3.6	10	2	1	0
JDM, Untreated	1	5	6.6+/−5.2	3	1	1	1
JDM, on Rx	4	15	10.0+/−3.3	15	4	0	0
JDM, off Rx	2	7	11.7+/−4.1	6	2	0	1

### EPC (CD34^+^/VEGFR2^+^/CD45dim^−^) number measurement by FACS

EPCs were identified with CD34^+^/VEGFR2^+^/CD45dim^−^ [14–16]. For each PBMC sample, at least 5 million cells were evaluated and incubated with a Live/Dead stain for 30 min at room temperature. The cells were washed and then divided into 4 tubes and blocked on ice with 2% Bovine Serum Albumin for 15 min. After washing, aliquots of the cells were stained according to the following: 1) no stain; 2) an isotype stain with IgG1-R-Phycoerythrin (PE); 3) a fluorescence minus one (FMO) stain with CD45-PB and CD34-APC; 4) and a stain with CD45-PB, CD34-APC, and VEGFR2-PE. Each tube was incubated for 30 min at room temperature, followed by two washes and fixation with 1% paraformaldehyde (PFA). The stained samples were analyzed by flow cytometry using the BD LSRFortessa instrument within 24 h. FlowJo software was used for data analysis, which was performed by first gating debris out of the light scatter plot; dead cells were excluded based on the Live/Dead stain. The live cells for the FMO sample were then analyzed on a plot of CD34-APC versus CD45-PB to view the CD34+ population of cells. The CD34+ cells were then analyzed on a plot of CD34-APC versus VEGFR2-PE; the CD34+/VEGFR2+ events were analyzed using the light scatter gate. The same method was applied to the stained sample, and the final CD34+/VEGFR2+ number for each patient was obtained after subtracting the CD34+/VEGFR2+ FMO number from the stained number.

### Statistical analysis

Correlations of the EPC numbers with other clinical and demographic variables, including DUD, DAS Skin (DAS SK), DAS Weakness (DAS WK), DAS Total Score, lipids and ERL were calculated using the Pearson correlation coefficient. Tests of associations of EPC with gender (boy vs girl), DUD, TNF-α-308A allele (GA/AA vs GG), vWF:Ag (normal vs abnormal based on ABO blood type), were performed using two-sample T- tests. The equality of mean EPC numbers between the 4 groups: control, untreated JDM, on medication JDM and off medication JDM was tested (ANOVA).

## Results

**Fig. 1:**
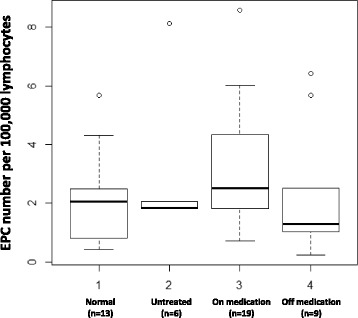
EPC (CD34^+^/VEGFR2^+^/CD45dim^−^) number measurements by FACS. PBMCs were stained with CD45-PB, CD34-APC, and VEGFR2-PE. The stained samples were analyzed by flow cytometry using the BD LSRFortessa instrument within 24 h. FlowJo software was used for data analysis. There were no significant differences between the data for any of the groups. Therefore, JDM have normal numbers of circulating EPCs, which differs from data reported for EPCs from adults with PM

Table 1 displays demographic data for the 13 healthy pediatric controls and 34 JDM patients. The EPC number was 2.28 ± 1.59/100,000 lymphocytes in healthy controls (Fig. 1, Bar 1), 3.13 ± 2.79/100,000 lymphocytes in untreated JDM (Fig. 1, Bar 2), 3.18 ± 2.03/100,000 lymphocytes in JDM on treatment (Fig. 1, Bar 3) and 2.29 ± 2.24/100,000 lymphocytes in JDM who had discontinued medication (Fig. 1, Bar 4). Although the EPC number was slightly higher in the untreated JDM (who were younger) than in healthy pediatric controls, it was not statistically significant (*p* = 0.55). There was no significant difference between the EPC number of controls and JDM taking medical therapy (*p* = 0.17) or between JDM who had improved and were taken off all medication (*p* = 0.99). ANOVA was used to test the equality of mean EPC numbers between the 4 groups — control, untreated, on therapy, and off therapy groups; there were no significant differences (*P* = 0.265). EPC number was not associated with DUD, ERL, DAS SK, DAS WK, age and cardiovascular risk factors such as LDL, HDL, triglyceride and cholesterol levels in JDM (Table 2). EPC number was not associated with gender, blood type, vWF:Ag levels, or the TNF-α-308A allele in JDM (Table 2).

**Table 2:** The association of endothelial progenitor numbers with clinical and laboratory variables

	*N*	Pearson correlation coefficient	*P*-value
DUD	34	0.0005	0.998
DAS SK	33	−0.1668	0.354
DAS WK	33	0.0874	0.629
ERL	34	0.0654	0.713
Age	34	−0.1533	0.387
LDL	22	0.1023	0.651
HDL	23	-0.0014	0.995
Cholesterol	23	−0.1768	0.420
Triglyceride	23	−0.0195	0.930
	*N*	EPC numbers (Mean+/−SD)	*P*-value
vWF:Ag	32	Abnormal: 3.84+/−3.00, *N* = 5 Normal: 2.72=/−2.05, *N* = 27	0.306
Gender	34	Boy: 2.32+/−0.78, *N* = 7 Girl: 3.03+/−2.39, *N* = 27	0.446
TNF-α -308A	34	GG:2.68+/12.06, *N* = 24 GA: 3.36+/−2.43, *N* = 10	0.409
Blood Type	28	A: 3.28+/−2.74, *N* = 14 O: 2.93+/−1.80, *N* = 14	0.693

*DUD* duration of untreated disease, *DAS SK* disease activity score skin, *DAS WK* disease activity score weakness, *ERL* end row loop, *LDL* low-density lipoprotein, *HDL* high-density lipoprotein, *vWF:Ag* von willebrand factor antigen

## Discussion

In this study, the number of EPCs in children with JDM was slightly increased, but not significantly different from healthy pediatric controls. Recently published data documented that the EPC number, as defined by CD133^+^ and CD34^+^, was decreased in adults with PM, but not DM [9], and decreased in adult RA [11], but not in adults with SLE [10]. However, when the EPCs from patients with SLE were quantified by per 10^6^ lymphocytes, they were decreased and the EPCs had a decreased proliferation rate, as well as increased apoptosis, impaired differentiation rate and reduced migratory capacity [10]. These results suggest that the reduction of EPC number and functionality might be a contributing factor to increased cardiovascular risk in adults with SLE and RA [10, 11]. In contrast, our pilot data did not document a significant difference in EPC number between healthy pediatric controls and JDM, treated or untreated. These data suggest that vascular damage in JDM may proceed by a pathway that differs from adults with PM, SLE, and RA, but may be similar to adults with DM. It does not answer the question, “Is JDM EPC function normal?” We could not safely obtain sufficient blood from the children to test this.

The age of the host is also a consideration. For example, miRNA-10a, which controls elements of the vascular system, was decreased in children with JDM, but not reported to be diminished in adults with DM [13]. Similarly, in Juvenile Idiopathic Arthritis (JIA) the number of circulating EPCs was in the normal range [17], as opposed to adult RA where the EPCs were decreased [11]. We used CD34^+^ and VEGFR2^+^ double positive biomarkers to assay progenitor endothelial cell numbers in children with JDM. The use of these markers is a more specific combination to identify EPC, because these double positive EPCs are both functionally intact and have the capacity for tube formation, both in vitro and in vivo [6]. As noted above, CD133 is present not only on EPCs, but also on numerous epithelial, hematopoietic, and various cancer stem cells; therefore, CD133 might be a less specific biomarker for EPCs [8, 18]. This lack of CD133 specificity could contribute to the increased EPC number in the FACS analysis of adult DM [9].

This pilot study supports the hypothesis that EPCs in children with JDM differ from adult PM, but might be similar to adult DM. Emerging data has identified differences in JDM children compared with DM adults with respect to dysregulation of microRNAs [13] and cytokine display [19], but EPC number does not appear to be in that category. It is axiomatic that the identification of the critical variables used to define disease specificity as well as age specificity is essential to achieve an understanding of the variations in the pathophysiology of the inflammatory myopathies.

## Abbreviations

DAS SK: Disease activity score, skin
DAS WK: Disease activity score, weakness
DAS: Disease activity score
DM/PM: Dermatomyositis and polymyositis
DUD: Duration of untreated disease
EPC: Endothelial progenitor cells
ERL: End row loop
FACS: Fluorescence activated cell sorting
FMO: Fluorescence minus one
HDL: High-density lipoprotein
JDM: Juvenile Dermatomyositis
JIA: Juvenile idiopathic arthritis
LDL: Low-density lipoprotein
PBMC: Peripheral blood mononuclear cells
PE: Phycoerythrin
PFA: Paraformaldehyde
RA: Rheumatoid arthritis
SLE: Systemic lupus erythematosus
vWF:Ag: von Willebrand factor antigen

## References

[1] Banker B, Victor M (1966). Dermatomyositis (systemic angiopathy) of childhood. Medicine (Baltimore).

[2] Bohan A, Peter JB (1975). Polymyositis and dermatomyositis (first of two parts). N Engl J Med.

[3] Bode RK, Klein-Gitelman MS, Miller ML, Lechman TS, Pachman LM (2003). Disease activity score for children with juvenile dermatomyositis: reliability and validity evidence. Arthritis Rheum.

[4] Christen-Zaech S, Seshadri R, Sundberg J, Paller AS, Pachman LM (2008). Persistent association of nailfold capillaroscopy changes and skin involvement over thirty-six months with duration of untreated disease in patients with juvenile Dermatomyositis. Arthritis Rheum.

[5] Greenberg SA, Pinkus JL, Pinkus GS, Burleson T, Sanoudou D, Amato AA (2005). Interferon-alpha/beta-mediated innate immune mechanisms in dermatomyositis. Ann Neurol.

[6] Asahara T, Murohara T, Sullivan A, Silver M, van der Zee R, Li T, Isner JM (1997). Isolation of putative progenitor endothelial cells for angiogenesis. Science.

[7] Grisar JC, Haddad F, Gomari FA, Wu JC (2011). Endothelial progenitor cells in cardiovascular disease and chronic inflammation: from biomarker to therapeutic agent. Biomark Med.

[8] Peichev M, Naiyer AJ, Pereira D, Zhu Z, Lane WJ, Williams M (2000). Expression of VEGFR-2 and AC133 by circulating human CD34(+) cells identifies a population of functional endothelial precursors. Blood.

[9] Ekholm L, Kahlenberg JM, Barbasso Helmers S, Tjärnlund A, Yalavarthi S, Kaplan MJ (2016). Dysfunction of endothelial progenitor cells is associated with the type I IFN pathway in patients with polymyositis and dermatomyositis. Rheumatology (Oxford).

[10] Ebner P, Picard F, Richter J, Darrelmann E, Schneider M, Strauer BE (2010). Accumulation of VEGFR-2+/CD133+ cells and decreased number and impaired functionality of CD34+/VEGFR-2+ cells in patients with SLE. Rheumatology (Oxford).

[11] Herbrig K, Haensel S, Oelschlaegel U, Pistrosch F, Foerster S, Passauer J (2005). Endothelial dysfunction in patients with rheumatoid arthritis is associated with a reduced number and impaired function of endothelial progenitor cells. Ann Rheum Dis.

[12] Niewold TB, Kariuki SN, Morgan GA, Shrestha S, Pachman LM (2009). Elevated serum interferon-alpha activity in juvenile Dermatomyositis: associations with disease activity at diagnosis and after thirty-six months of therapy. Arthritis Rheum.

[13] Xu D, Kachaochana A, Morgan GA, Vanin EF, Huang CC, Pachman LM (2016). MicroRNA-10a regulation of Proinflammatory mediators: an important component of untreated juvenile Dermatomyositis. J Rheumatol.

[14] Khan SS, Solomon MA, Philip McCoy J (2005). Detection of circulating endothelial cells and endothelial progenitor cells by flow cytometry. Cytometry B Clin Cytom.

[15] Colombo E, Marconi C, Taddeo A, Cappelletti M, Villa ML, Marzorati M (2012). Fast reduction of peripheral blood endothelial progenitor cells in healthy humans exposed to acute systemic hypoxia. J Physiol.

[16] Bui KC, Weems M, Biniwale M, George AA, Zielinska E, Azen CG (2013). Circulating hematopoietic and endothelial progenitor cells in newborn infants: effects of gestational age, postnatal age and clinical stress in the first 3 weeks of life. Early Hum Dev.

[17] Obeid J, Nguyen T, Cellucci T, Larche M, Timmons BW (2015). Effects of acute exercise on circulating endothelial and progenitor cells in children and adolescents with juvenile idiopathic arthritis and healthy controls: a pilot study. Pediatric Rheumatol.

[18] Yoder MC (2012). Human endothelial progenitor cells. Cold Spring Harb Perspect Med.

[19] López De Padilla CM, Crowson CS, Hein MS, Pendegraft RS, Strausbauch MA, Reed AM (2017). Gene expression profiling in blood and affected muscle tissues reveals differential activation pathways in patients with new-onset juvenile and adult dermatomyositis. J Rheumatol.

